# Modeling and Simulating Complex Conflict Management Using Reaction Networks

**DOI:** 10.3390/e28070754

**Published:** 2026-07-01

**Authors:** Tomas Veloz, Dirk Bruin, Cedric De Coning

**Affiliations:** 1 Departamento de Matemáticas, Universidad Tecnológica Metropolitana, Santiago 833038, Chile; 2 Center Leo Apostel for Interdisciplinary Studies, Vrije Universiteit Brussel, 1160 Brussels, Belgium; dirkpbruin@gmail.com; 3 Norwegian Institute of International Affairs, 0160 Oslo, Norway; cdc@nupi.no

**Keywords:** Lake Chad Basin, protracted conflicts, complex adaptive systems, peace-building, reaction networks, Chemical Organization Theory, social organization, Service’s typology

## Abstract

Evidence suggests that protracted conflicts persist because several forms of socio-political organization run simultaneously on the same population, resources, and territory. Reading Service’s typology of bands, tribes, chiefdoms, and states not as evolutionary stages but as coexisting and superposed social organizations, we model conflict as a reaction network where each social form is a self-maintaining set of stocks—a chemical organization—and conflicts arise where competing productive logics between organizations generate stocks with negative connotation, such as grievances and displacement. Taking the Lake Chad Basin as inspiration, we build a ladder of progressively richer models arriving a mixed chiefdom–state configuration compatible with current views on the conflict. As the model complexifies, kinetic approaches become uninformative; we therefore develop complementary stoichiometric methods that are parameter-free and thus are far easier to measure and compute. These diagnostics reveal a structural bias toward conflict: transitions into conflict regimes are systematically richer than transitions out. We show how a dual chiefdom–state form acts as a conflict attractor within a closed conflict–peace loop that transits among documented different forms of organization. Conflict management then becomes the identification of the mechanisms that redirect rather than change the state of a self-sustaining organization—here, elite-surplus redistribution—and of the timescales at which such redirection is observable, turning intervention design into a structural rather than a parameter-tuning problem.

## 1. Introduction

The Lake Chad Basin insurgency has caused over 350,000 deaths and displaced more than three million people since 2009 [1,2]. In May 2021, the leader of one Boko Haram faction was killed by a rival; the war’s actor inventory was reshuffled, yet the war continued [3,4]. The same resilience recurs across protracted conflicts—Colombia, the eastern Democratic Republic of Congo, Yemen, Afghanistan—where changes of leadership, doctrine, or dominant party leave the conflict system structurally intact [5,6,7]. Why do these systems persist?

Existing formal frameworks struggle with this question because they treat conflict as a contest between two camps, each a unit with stable boundaries and a single organizational logic [8,9,10]. Lake Chad refuses that template: the Islamic State West Africa Province (ISWAP) taxes and adjudicates like a quasi-state [1,11,12], the Bakura faction sustains itself through predation [3], while vigilante formations, emirate authorities, pastoralist communities, and displaced populations each operate on a different logic—all on the same population, resources, and territory. The conflict is in part a contest among these coexisting forms.

We formalize this by reading Service’s typology of socio-political organization—band, tribe, chiefdom, state [13,14,15,16]—not as evolutionary stages but, following its non-linear interpretation [17], as a vocabulary for forms that coexist and interconvert. We render each form in the language of reaction networks [18,19]: a self-maintaining set of transformations—a chemical organization [20]—that regenerates its own conditions. Conflict persists, on this account, for the same structural reason living systems persist.

The paper makes three formal claims and one methodological one. (1) Each regime form (tribe, chiefdom, state) is a chemical organization, and the forms are partially ordered by inclusion. (2) A single organization admits multiple self-maintaining modes of operation—most clearly, the redistributive versus predatory chiefdom. (3) The mixed chiefdom–state configuration matching Lake Chad is itself an organization whose several modes jointly sustain the conflict—the computable counterpart of de Coning’s “self-sustaining path dependencies of violence” [7,21]. Methodologically, as the network grows from 10 to 37 reactions, kinetic description becomes intractable; we therefore develop parameter-free structural diagnostics, each introduced when dynamics stops discriminating. Their headline result is a structural bias toward conflict: transitions into conflict regimes are systematically richer than transitions out.

The paper is organized as follows. Section 2 reviews existing modeling traditions and argues for a process ontology; Section 3 reframes Service’s typology as a vocabulary for coexisting regimes; Section 4 introduces the reaction-network formalism and a minimal band–tribe model with its two dynamical regimes; Section 5 adds the chiefdom and develops the flow-, mode-, and timescale-based diagnostics; Section 6 adds the state and analyses the full network through Chemical Organization Theory and its transition geometry; and the Conclusions gather the methodological and managerial implications.

## 2. Conflict Modeling and the Need for a Process Ontology

A wide variety of formal frameworks has been brought to bear on conflict dynamics. Each captures part of what is needed; none, taken alone, supports the regime-coexistence reading we develop here. This section reviews the principal traditions, identifies what each contributes and what each lacks, and argues that what is missing is a *process ontology*—a modeling language whose fundamental units are transformations of conditions rather than interactions between fixed entities.

### 2.1. Modeling Traditions

*Differential-equation models* [8,10,22,23] give precise trajectories but treat actors as fixed entities, an assumption that fragments under factional splintering or the appearance of new actors. *Game-theoretic approaches* [9,24,25] analyze equilibria under fixed choice structures but struggle with conflicts that impose sustained costs on all parties [6] and whose actor inventory co-evolves with the interaction. *Agent-based models* [26,27] generate emergent macro-patterns from micro-rules but require many parameters and rarely yield closed-form results. *Dynamical-systems frameworks* [28,29,30] offer valuable concepts—attractors, basins, bifurcations—but remain largely metaphorical, falling back on ODEs or ABMs for implementation. *Network models* [31,32,33] capture relational structure but typically treat the graph as static rather than co-evolving with the conflict. *Causal-loop and stock-and-flow models* [28,34] make feedback visible, but their loops are qualitative: no formal criterion says which loops sustain which configurations, and no method isolates the minimal disruptive intervention. Table 1 summarizes the comparison.

### 2.2. The Gap and the Process-Ontology Requirement

Common to these traditions is a fundamental assumption: that the entities of analysis—states, groups, agents—have stable identities, and that what changes is their quantitative interactions. This assumption fails under exactly the conditions that define protracted conflict. In Lake Chad, the entity “Boko Haram” that existed in 2014 had largely dissolved by 2022, replaced by a configuration of ISWAP, Bakura, defectors, vigilantes, and shifting alliances; yet many of the underlying processes (recruitment from displaced populations, capture of local surpluses by armed elites, contestation over taxation rights, attacks on state infrastructure) continued operating across the changeover [3,12]. The processes outlasted the entities. Any framework whose ontological primitives are the entities cannot represent this fact directly; it must reconstruct it through ad hoc rules for entity birth, death, and identity-change.

A process ontology takes the opposite stance. The fundamental primitives are transformations—grievance into recruitment, recruitment into armed force, armed force into displacement, and displacement into more grievance—and the entities are reconceptualized as bundles of conditions transformed by, and stabilized through, these processes. Under this ontology a *regime form* is a self-sustaining pattern of transformations: a chiefdom is the set of transformations that capture surplus from a tribal base, recruit personnel from the displaced, and reproduce both an elite surplus and a chiefdom military. A state is a different self-sustaining pattern, drawing on infrastructure-mediated recruitment and legitimacy. Several such patterns can run on the same population at the same time, sharing some transformations and competing for others.

Table 1 summarizes the comparison.

Reaction networks supply such an ontology. Developed in biochemistry to model systems of transformations with self-maintaining cycles, their mathematics has been applied to ecology, social systems, and worldview dynamics [35,36,37]. The formalism is equivalent to Petri nets and vector addition systems, making a large body of theoretical results directly applicable [38].

The remainder of the paper develops the reaction-network apparatus and applies it to a model of conflict whose species correspond to regime forms (band–tribe–chiefdom–state) in a heterogeneous conflict system.

## 3. Service’s Typology Reframed: From Stages to Coexisting Regimes

### 3.1. Bands, Tribes, Chiefdoms, States

Elman Service’s *Primitive Social Organization* [13] and the subsequent *Origins of the State and Civilization* [14] introduced a four-fold classification of socio-political forms that underpins cultural anthropological theories. A *band* is a small kin-based group, typically twenty to one hundred individuals, with face-to-face relationships, no formal leadership positions, and minimal social stratification beyond age and gender. A *tribe* is a larger settlement-based or seasonally mobile community of several hundred to several thousand, organized through reciprocal exchange between kin segments and with leadership roles—most famously the “Big Man” described by Sahlins [39]—that emerge from personal capacity and prestige rather than from formal office. A *chiefdom* is a hierarchical polity unified by a hereditary chief, integrated through ranked descent groups, supported by a class of elites, and characterized by chiefly redistribution of surplus and the beginnings of specialized roles, including in some cases organized armed force [40,41]. A *state* is an institutionalized polity with codified law, professional administration, monopolistic claims on the legitimate use of force, and infrastructure that enables the production of legitimacy as a reproducible resource [14,42].

The four forms differ along several structural dimensions that motivate our reaction network model: the existence and form of accumulated surplus (no surplus in the band, communal surplus in the tribe, elite-controlled surplus in the chiefdom, state-controlled surplus in the state); the organization of force (no specialized armed body in the band or tribe, chiefdom military in the chiefdom, state military in the state); the basis of authority (egalitarian and informal in the band and tribe, hereditary and ranked in the chiefdom, institutional and legitimacy-based in the state); and the relation to the displaced (the band has no mechanism for absorbing them other than kin reintegration, the tribe absorbs through stored surplus, the chiefdom recruits them as armed force, the state recruits them through infrastructure-mediated channels).

### 3.2. From Stages to Coexisting Forms

Service’s typology has been criticized, with substantial justification, for the unilinear evolutionary scheme it has been read into—bands becoming tribes becoming chiefdoms becoming states, in a single ordered ascent [43,44]. Yoffee [43] argues that many archaic states did not pass through identifiable chiefdom stages and that “chiefdom” as a category collects polities so heterogeneous as to obscure more than it reveals. Pauketat [44] extends the critique. Wengrow and Graeber [17] mount the most forceful version: many of the world’s documented societies have moved between forms in both directions and have held several forms simultaneously across different domains of life—one form for ritual, another for war, and another for everyday subsistence. The unilinear reading, they argue, was never warranted by the ethnographic record; it was projected onto it.

Our purpose here is not to defend Service’s typology against the linearity critique by reading it more cautiously, but to formalize the multidirectional reality the critique points to while keeping the vocabulary the typology supplies. Chemical Organization Theory is precisely the framework for this. Its central object is not a single organization but the *lattice of organizations*, partially ordered by species-set inclusion, and its central dynamical claim is that a reaction system, under perturbation, can move between organizations in any direction the network’s reactions permit [20,45,46]. In a biochemical system, a perturbation can collapse a complex organization to a smaller one (the system loses species and capacities) just as readily as another perturbation can build up a larger organization from a smaller one. There is no built-in arrow from simple to complex, only the structural constraint that transitions follow the lattice. The same mathematics, applied to socio-political organizations, gives us exactly the bidirectional, multi-form picture that Wengrow and Graeber argue the ethnographic record requires: a state can collapse into a configuration of chiefdoms, a chiefdom can dissolve into kin-based tribes, a stressed tribe can fragment into stressed bands, and movement in the reverse direction is equally possible—each transition corresponding to a structural perturbation that adds or removes species and reactions.

### 3.3. Lake Chad Basin Through the Typology

The Lake Chad Basin, encompassing parts of northeastern Nigeria, far north Cameroon, western Chad, and southeastern Niger, exhibits all four Service forms simultaneously. We describe the actor inventory through the typology to fix intuition for the formal model.

- Bands: The 3.4 million internally displaced persons across the Lake Chad Basin [2] have been detached from the surplus structures and reciprocal networks that previously stabilized them, while many remain organized in family and clan units, their position with respect to the political economy is band-like: they lack the capacity to produce or store communal surplus, depend on humanitarian assistance or precarious labour, and are exposed to recruitment by chiefdom-form armed actors.
- Tribes: Settled agrarian communities and pastoralist groups across the basin operate principally through tribal logics: kin-based segmentary organization, reciprocal labor exchange, customary dispute resolution by elders, and seasonal redistribution practices that accumulate as a community resilience reservoir. Big Man leadership—influential elders, lineage heads, prosperous traders—is widespread and unstable in the way Sahlins described.
- Chiefdoms: Several distinct chiefdom-form polities operate in the region. Traditional emirate authorities in northern Nigeria and Cameroon retain governance functions that pre-date and partially overlap with the modern state. The CJTF and other vigilante formations are chiefdom-form polities of recent origin, with hereditary or quasi-hereditary leadership, organized armed capacity, and contested legitimacy [47,48]. Most importantly for our analysis, the JAS/Bakura faction operates as a predatory chiefdom: “at the crossroads between sectarianism, predation and clientelism” [3], with chiefly authority, organized armed force, and surplus extraction without redistribution.
- States: The four national states (Nigeria, Cameroon, Chad, Niger) and their joint security architecture (the Multi-National Joint Task Force and the RS-SRR coordination framework) operate as state-form polities, with variable depth of presence: substantial in major cities, attenuated in rural peripheries, contested in areas of ISWAP and JAS activity. International humanitarian and stabilization actors (UN agencies, regional bodies, foreign donors) supply external resources to these states and, indirectly, to chiefdom-form actors as well.

Conflicts are most accurately described not as a war between two parties but as a contest among several coexisting regime forms over the same population. In the case of the Lake Chad Basin, territory, and resource base, with cross-regime transformations—corruption, co-optation, predation, and foreign aid—linking them. How to model these kinds of configurations and analyze their mutual operation to identify self-sustaining features in the Lake Chad Basin conflict is the formal question we analyze. Notably, ISWAP occupies a position that operates as a chiefdom but operates with state form aspirations, providing taxation, adjudication, public services, and a degree of institutional differentiation in territories it controls. The empirical material on ISWAP describes it variously as a “proto-state”, a “quasi-state actor”, and an “alternative state” [1,11,12]. Within our typology this case is best understood as a coexistence between state and chiefdom, where the chiefdom replaces significant structures that the state-form is not able to protect (a chain of command for resource allocation, codified rules, infrastructure investment, and recruitment through legitimacy as well as through coercion) without yet attaining the socio-political legitimacy and infrastructure base of an established state.

## 4. Reaction Network Modeling of Conflicts

### 4.1. Reaction Networks: A Language for Collective Transformation

Reaction networks were developed in biochemistry to model metabolic phenomena such as productive circuits and self-production, but their mathematical structure is domain-independent [49]. A reaction network is formally defined as a pair (M,R), where $M=\{s_{1},s_{2},\ldots ,s_{n}\}$ is a finite set of *species* and $R=\{r_{1},r_{2},\ldots ,r_{m}\}$ is a finite set of *reactions*. Each reaction $r_{i}$ is a transformation rule of the form

(1) $$\alpha_{i1}s_{1}+\ldots +\alpha_{in}s_{n}\;⟶\;\beta_{i1}s_{1}+\ldots +\beta_{in}s_{n},$$

where $\alpha_{ij}$ and $\beta_{ij}$ are non-negative stoichiometric coefficients representing the quantities of species $s_{j}$ consumed and produced by reaction $r_{i}$. Unlike domain-specific models that assume particular interaction mechanisms, reaction networks provide a process-oriented ontology where the fundamental building blocks are *transformations* rather than entities [50,51]. The *stoichiometric matrix S* is the central analytical object: an n×m matrix whose entry $S_{ij}=\beta_{ij}-\alpha_{ij}$ encodes the net change in species $s_{i}$ per firing of reaction $r_{j}$. A *process vector* $v\in R_{\geq 0}^{m}$ specifies how much each reaction fires in a given time interval, so the net state change is

(2) Δx=S·v.

The process v specifies how many times each of the possible reactions occurs, but it does not specify a time interval for it, and the stoichiometric matrix is time-independent by definition. Therefore, Equation (2) characterizes a broad space, named Δx, of *possible variations* the system’s state x can undergo. Any further specification on how reactions occur will instantiate one possibility.

### 4.2. Conflicts as Reaction Networks

Conflict systems fit naturally into the reaction network language. The entities involved—combatants, institutions, resources, grievances, trust—participate in transformations that change their quantities and qualities: grievances mobilize into violence, violence erodes institutions, institutions generate trust, and trust rebuilds institutions. Coleman’s dynamical systems approach these variables as “stocks”—accumulated quantities that feed and drain each other through coupled flows [28,29]. This stock-and-flow perspective captures an important structural feature of conflict: persistence is driven by the mutual reinforcement of multiple accumulated conditions, not by any single cause. Reaction networks generalize this perspective: stocks become species, flows become reactions, and stoichiometric coefficients quantify the transformation ratios.

Stocks range from objectively countable (combatants, weapons, territory, finance) through semi-tangible proxies (economic output, institutional capacity) to subjective quantities (grievance, trust, legitimacy) estimated via interviews or online activity [52,53,54].

We introduce the concept of *mechanisms of stock transformation* as the natural unit of translation between what is known about the conflict and our reaction network modeling of it. A mechanism of stock transformation is a structured narrative that specifies which stocks participate in a given transformation and what role each plays—requirement, catalyst, output. These mechanisms are the way analysts, practitioners, and communities already describe conflict: as accounts of how one condition produces or depletes another, under what circumstances, and with what consequences for other conditions. Translating a mechanism into a reaction requires identifying the consumed species (drivers depleted by the transformation), the produced species (outputs generated), the catalytic species (enablers present but not depleted), and an estimation of the stoichiometric ratios (how many units of each are involved).

The dynamics of a reaction network model require a specification of how reactions occur over time, i.e., a kinetic rule. Unlike chemical kinetics, conflict reactions vary in controllability—from those outside any policy reach (climate cycles, social forgetting, violence-driven migration), through those only partially steerable (local violence events), to those directly implementable as policy (investment, governance, community programs, security operations).

Whether a particular conflict configuration persists is thus a question about the collective behavior of its mechanisms of stock transformation: do they jointly form a self-producing structure that continuously regenerates its own conditions of existence? De Coning’s “self-sustaining path dependencies of violence” [21] meet this condition. This is the bridge between descriptive conflict analysis and formal analysis that the reaction network framework provides.

### 4.3. A Minimal Band–Tribe Model and Its Dynamical Realizations

As a start, we construct a minimal reaction network to represent the stressed band-tribe described in Section 3.

Species in our reaction network are partitioned into three functional groups:

- **Population strata** (people in different organizational positions):
  - *C*: *Collective* people—those embedded in a band-tribe with reciprocal labor and access to communal surplus;
  - *X*: *Displaced* people—those detached from the surplus and reciprocity structures of *C*;
- **Resource stocks** (the same underlying surplus, organized under different logics):
  - Res: ambient resources—food, land, water flows that enter the system from the environment;
  $C_{\mathrm{Res}}$: *community resilience*—surplus stored and redistributed under tribal logic (the Big Man stock);
- **Conflict marker:**
  - *G*: *grievance*—accumulated tension produced from social disruptions related to absence of resources or violence.

For the analyses that follow we group these into two marker sets:

(3) $$M_{\mathrm{community}}=\left\{C,\;C_{\mathrm{Res}}\right\},\;M_{\mathrm{conflict}}=\left\{X,\;G\right\},$$

with Res acting as a transverse substrate of both marker sets.

We introduce eleven reactions $R=\{r_{0},\ldots ,r_{10}\}$ in three functional blocks and described in Table 2; a brief narrative follows.

*Environmental input.* A single reaction $r_{0}$ supplies ambient resources at a constant rate, representing climate, land productivity, and external inflows.

*Band–tribe block ($r_{1}$–$r_{7}$).* The collective *C* consumes ambient resources to subsist ($r_{1}$); when $C_{\mathrm{Res}}$ is present, the collective produces additional community resilience ($r_{2}$, autocatalytic in $C_{\mathrm{Res}}$); stored resilience can be redistributed back into ambient stock ($r_{3}$). Members of the collective become displaced under stress ($r_{4}$); displaced people return to the collective when community resilience is available to absorb them ($r_{5}$). Both ambient resources and community resilience decay autonomously ($r_{6}$, $r_{7}$).

*Conflict block ($r_{8}$ and $r_{9}$).* Direct competition between displaced and collective people over ambient resources produces grievance; accumulated grievance displaces members of the collective ($r_{9}$, second-order in *G*).

Before introducing dynamics, we visualize the stoichiometric matrix *S* of our model. The matrix has dimensions 5×11 (five species: $C,X,\mathrm{Res},C_{\mathrm{Res}},G$; ten reactions: $r_{0}$ through $r_{10}$). Table 3 displays *S* with catalytic entries—those species that participate in a reaction without net consumption or production (appearing on both sides)—marked in blue.

The catalytic species—*C* in $r_{1}$ and $r_{2}$ (the collective sustains itself and rebuilds resilience), and *X* and *C* in $r_{8}$ (grievance is generated from the interaction of displaced and collective, but their presence is required to sustain the process)—are crucial to understanding feedback loops. Catalytic species enable reactions without being depleted, making them natural bottlenecks and control points in the system’s dynamics.

The stoichiometric matrix specifies which state changes are structurally possible (Equation (2)). Determining *which regime* the system enters requires specifying the kinetic rules—the rates at which each reaction proceeds as a function of the current state. We employ two complementary simulation methods:

Deterministic (ODE):

The state evolution is governed by

(4) $$\frac{dx}{dt}=S\;\cdot \;v\left(x;\theta \right),$$

where the process vector $v\left(x;\theta \right)\in R_{\geq 0}^{m}$ collects all reaction rates. Reaction rates without saturation follow the mass-action kinetics

(5) $$v_{i}\left(x;k_{i}\right)=k_{i}\prod_{j}x_{j}^{\alpha_{ij}},$$

where $k_{i}$ is the reaction’s rate constant. and rates of reactions with saturation (see Table 2) is assigned follow Michaelis–Menten kinetics:

(6) $$v_{i}\left(x;k_{i},K_{i}\right)=k_{i}\frac{\prod_{j}x_{j}^{\alpha_{ij}}}{x_{j}+K_{i}},$$

where $k_{i}$ is the maximal rate constant and $K_{i}$ represents saturation thresholds. The kinetic parameters $k_{i}$ and $K_{i}$ collectively form a high-dimensional space. Under the same initial conditions different on these parameters lead to different behaviors.

Stochastic (Tau-Leaping)

The stochastic formulation treats species abundances as discrete, non-negative integer populations, $x\in Z_{\geq 0}^{n}$, appropriate for conflict communities at the band–tribe scale where N∼10–100 individuals per category. Reactions fire in discrete bursts over a fixed time step τ. The number of times reaction *j* fires in [t,t+τ) is drawn independently as

$$n_{i};∼;\mathrm{Poisson}!\left(\lambda_{i}\left(x\right),\tau \right),$$

where $\lambda_{i}\left(x;\kappa_{i}\right)=\kappa_{i}\pi_{i}\left(x\right)$. $\pi_{i}\left(x\right)$ is the propensity (expected firings per unit time) and $\kappa_{i}$ is the stochastic rate constant. The propensity replaces the ODE product kinetics with the enabling degree

$$\pi_{i}\left(x\right)=\min_{j:\alpha_{ij}>0}\left\{\frac{x_{j}}{\alpha ij}\right\},$$

where $\alpha_{ij}$ denotes the reactant stoichiometric coefficient of species *j* in reaction *i*. The enabling degree measures how many simultaneous firings of reaction *i* the current state can sustain: it is bounded by the most depleted stoichiometric participant—the bottleneck reactant. Catalytic species enter $\pi_{i}$ as enabling constraints even though they are not consumed.

The state update at each step is

$$x\left(t+\tau \right)=x\left(t\right)+Sn\left(t\right),\;n_{i}\left(t\right);\leq ;\left\lfloor \pi_{i}\left(x\left(t\right)\right)\right\rfloor ,$$

where the bound $\left\lfloor \pi_{i}\right\rfloor$ prevents any reaction from over-consuming its reactants in a single step. When multiple reactions draw simultaneously from the same species pool—a contention event—their firing counts are scaled down proportionally until the updated state remains non-negative for all species.

The stochastic rate constants $\kappa_{i}$ are parameterized independently of the ODE constants $k_{i}$: since $\pi_{i}$ (a minimum over quotients) replaces the multiplicative product $\prod_{s}x_{j}^{\alpha_{ij}}$—and the Michaelis–Menten denominator where applicable—their numerical values differ. At initial conditions $x_{0}$, the ratio $v_{i}\left(x_{0}\right)/\pi_{i}\left(x_{0}\right)$ provides a guideline for matching the two parameterizations, but they diverge as the system evolves due to the fundamentally different functional forms.

### 4.4. Simulating Two Regimes in the Basic Model

The Regional Strategy for the Stabilization, Recovery, and Resilience of the Lake Chad Basin (RS-SRR) and recent syntheses of basin fieldwork [55] converge on a single diagnostic question: under what conditions does the community substrate regenerate the stored surplus and reciprocity structures (kin networks, customary dispute resolution, seasonal redistribution) that absorb the displaced, and under what conditions does this regeneration fail? As a proof of concept, the band–tribe network in Table 2 is the smallest reaction network in which this question admits a formal answer.

We simulate the network under two parameter configurations from the same initial state $\left(C,X,\mathrm{Res},C_{\mathrm{Res}},G\right)=\left(10,3,5,2,1\right)$ (full constants in Supplementary Materials). The configurations differ in two multipliers: $\alpha_{s}$ (*solidarity*), scaling surplus formation ($r_{2}$) and reintegration ($r_{5}$); and $\alpha_{r}$ (*reactivity*), scaling grievance generation ($r_{8}$) and violent displacement ($r_{9}$).

We provide all the details to compute our simulations and other calculations in the Supplementary Material. Moreover, a GitHub repository contains the reaction network and scripts to run the simulations (https://github.com/tveloz/pyCOT/tree/master/projects/XCEPT_Article_1) (accessed on 14 June 2026).

**Figure 1:**
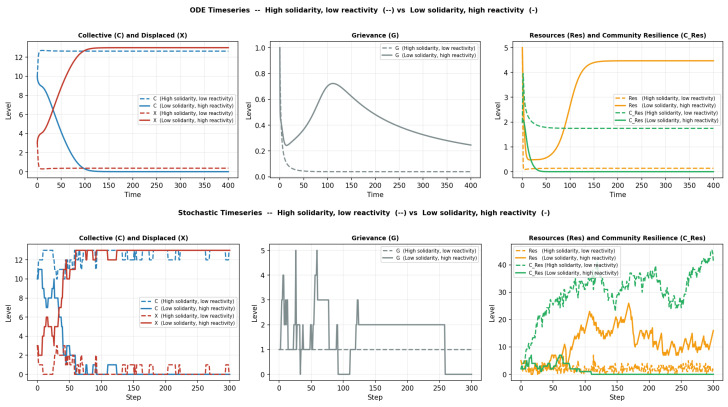
**Top**: ODE Timeseries for the basic model under Regime I (dashed) and Regime II (solid). **Down**: Stochastic (τ-leaping) timeseries for the same two regimes. Shaded bands: spread across five independent realizations. Species colors: *C* (blue), *X* (red), Res (orange), $C_{\mathrm{Res}}$ (green), *G* (gray).

**Regime I—high solidarity, low reactivity ($\alpha_{s}=2.0$, $\alpha_{r}=0.5$).** The ODE solution converges to *C*-majority, sustained $C_{\mathrm{Res}}$, low *X*, and negligible *G* (Figure 1 left, dashed). Stochastic realizations fluctuate around this attractor (Figure 1, dashed). The community sustains itself.
**Regime II—low solidarity, high reactivity ($\alpha_{s}=0.3$, $\alpha_{r}=3.0$).** Suppressed surplus growth and reactive displacement combine: grievance accumulates through (X,C,Res) contacts, the quadratic feedback in $r_{9}$ drives a violence wave, and $C_{\mathrm{Res}}$ collapses before reintegration can operate. The system settles into an *X*-dominated attractor with no recovery pathway open (Figure 1 solid). This is the collapsed-community configuration the RS-SRR explicitly seeks to prevent [55].

Both regimes arise from the same stoichiometry, distinguished only by two kinetic multipliers. In the basic model the two outcomes are readable from the stocks themselves. However, it is important to notice that this kind of work does not scale well when more variables are incorporated. The chiefdom extension that follows produces qualitatively distinct regimes that dynamical systems analysis make already very difficult to discriminate, motivating stoichiometric methods that are parameter-free.

## 5. Complementing Dynamical Analysis with Stoichiometric Methods

Following Service’s typology, we extend the basic network with a chiefdom—a hierarchical group *H* that is seeded as a structural perturbation [50,51].

The reactions in the extended model are based on empirical record on the Lake Chad Basin, which places multiple hierarchical formations on the same population. Foucher and El Hadji [3] contrast the Islamic State West Africa Province (ISWAP), with its “streamlined, bureaucratic governance that limits the amount of violence committed against Muslim civilians,” with the Bakura faction of JAS, which “resists this rationalization… at the crossroads between sectarianism, predation and clientelism.” The Civilian Joint Task Force and traditional emirates operate as further hierarchical formations [11,12]. The rebel-governance literature has developed a parallel typology, distinguishing extensive governance (taxation paired with public goods provision and dispute resolution) from limited governance (security only, with predation) [56,57,58]. Our model encodes two chiefdom strategies (*protection*, *exploitation*) as opposed modes of operation of the same chiefdom species, and additionally explores the influence of foreign aid. Four questions, each grounded in a distinct scholarly conversation, structure the analysis:

**Q1. Does a hierarchical authority integrate or exploit the community it sits on top of?** The rebel-governance literature has documented that armed authorities can either substitute for absent state functions or extract surplus and amplify displacement [3,56].
**Q2. Does external aid reach the population as intended?** Anderson’s *Do No Harm* [59] and subsequent empirical work on elite capture [60] established that aid delivered through host-country authority is never neutral with respect to conflict dynamics.
**Q3. Why can a single change in a program make all the difference?** Complexity-informed peacebuilding [7] and the systems literature on leverage points [61] argue that intervention impact is not proportional to investment: rules determining where flows go can have larger effects than changes in flow volumes.
**Q4. Why does the situation look different at different timescales?** Adaptive peacebuilding requires monitoring at multiple horizons because short-window observation captures noise and long-window observation captures structure, and the two regularly give opposite verdicts [7,55,62].

We take up each question: while we introduce progressively parameter-free stoichiometric methods: Q1 in Section 5.2 (stocks), Q2 and Q3 in Section 5.3 (flows), and Q4 in Section 5.4 and Section 5.5 (timescale).

### 5.1. The Chiefdom + Aid Extended Model

The chiefdom extension adds four species ($H_{\mathrm{Res}}$, $P_{H}$, $F_{H}$) and eleven reactions ($r_{11}$–$r_{21}$); the state vector becomes $\left(C,X,H,\mathrm{Res},C_{\mathrm{Res}},H_{\mathrm{Res}},G,P_{H},F_{H}\right)$. Table 4 summarizes the new reactions and the strategy multipliers $a_{j}^{\mathrm{prot}},a_{j}^{\mathrm{expl}}$ that distinguish the two modes. The chiefdom redistributes effort across eight controllable reactions $\left\{r_{12},r_{13},r_{15},r_{16},r_{17},r_{18},r_{19},r_{20}\right\}$ through a strategy vector $a\in R_{\geq 0}^{8}$, which multiplies the stochastic propensities, $\lambda_{j}^{\mathrm{eff}}\left(x;a\right)=a_{j}\;\kappa_{j}\;\pi_{j}\left(x\right)$, where $\pi_{j}$ is the enabling degree of Section 4.4. Non-controlled reactions run at $a_{j}=1$.

### 5.2. A View on the Kinetics to Ground the Use of Stoichiometric Tools

Before introducing the stoichiometric tools, we will illustrate how the dynamics of the incorporation of Chiefdom into our model looks like. In order to analyze question Q1, we consider two strategies: One based on protection, and another based on exploitation. In Table 4, we represent in columns $a_{j}^{\mathrm{prot}}$ and $a_{j}^{\mathrm{expl}}$ how the strategies modify the reactions propensities in our stochastic dynamics. Additionally, we consider cases with and without foreign aid separately. This distinction is important because in the former case the Chiefdom is able to operate resources independently of its interaction with the community.

**Figure 2:**
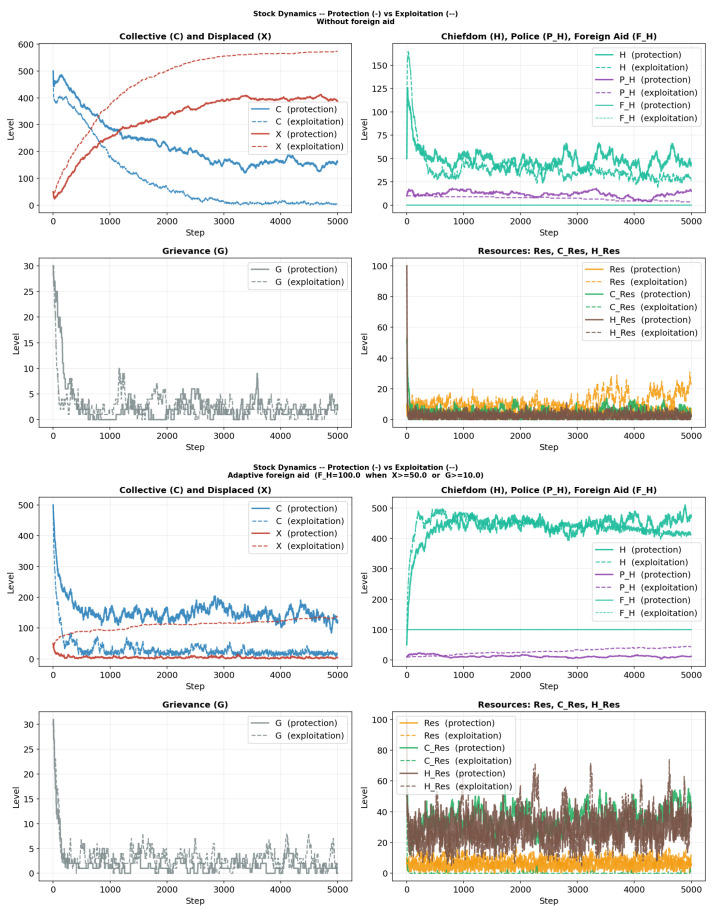
Stocks trajectories: without aid (**left**), with unconditional aid (**right**). Populations and resource stocks for protection (dashed) and exploitation (solid). Solid lines: one realization; bands: ±1 s.d. across 50 bootstrap resamplings.

In Figure 2, we illustrate the results of our the simulations. Results enable answering **Q1**. For simplicity, we will assume that *H* integrates the community when H>C>X, and *H* exploits the community when X>H and X>C.

By looking at Figure 2, we observe that the integration/exploitation dichotomy—imposed by how Q1 is formulated—is not sufficient to describe the model results because the possibility of foreign aid complexifies the landscape of scenarios obtained:

The four regimes (Table 5) differ by which reactions dominate. With aid, protection yields *integration* (H>C>X): $P_{H}$ suppresses grievance and $r_{18}$ reinvests $H_{\mathrm{Res}}$ in $C_{\mathrm{Res}}$ so the collective is subordinate but not displaced; exploitation yields *expulsion* (H>X>C): aggressive capture ($r_{12}$) outpaces reintegration ($r_{5}$). Without aid, protection settles into *adaptation* (H∼C) as $C_{\mathrm{Res}}$ still readmits the displaced and grievance decays, while exploitation *collapses* (X>H>C): extraction fuels grievance, $r_{9}$ multiplies displacement, and the chiefdom cannot sustain $P_{H}$. We summarize the results in Table 5.

The results we obtained are dependent on our parametric choice. Note that having 22 reactions, nine species, we obtain roughly twenty kinetic constants, eight strategy multipliers, and an aid level. In general it is very difficult to know the correct parameters for a conflict model like this. Therefore, the aim of this section is to show how can we complement dynamical analysis with methods that do not depend directly on the kinetic information, but only on the reaction network’s structure.

### 5.3. Stock Flows and Modes of Operation

In order to advance a better understanding of how the parameters and strategies influence the dynamics, we will analyze more carefully **Q2** by looking at how the stock $H_{\mathrm{Res}}$ flows through the different strategies. Namely, we define *Redistribution/peacekeeping* as the total of $H_{\mathrm{Res}}$ used to fire $r_{16}$ plus $r_{18}$. Note that $r_{18}$ requires 2 species of type $H_{\mathrm{Res}}$ so this reaction counts twice per firing. Similarly, we define *Elite expansion* by the firing of $r_{15}$, and *Other maintenance* by the firings of $r_{11}$ plus $r_{17}$.

**Figure 3:**
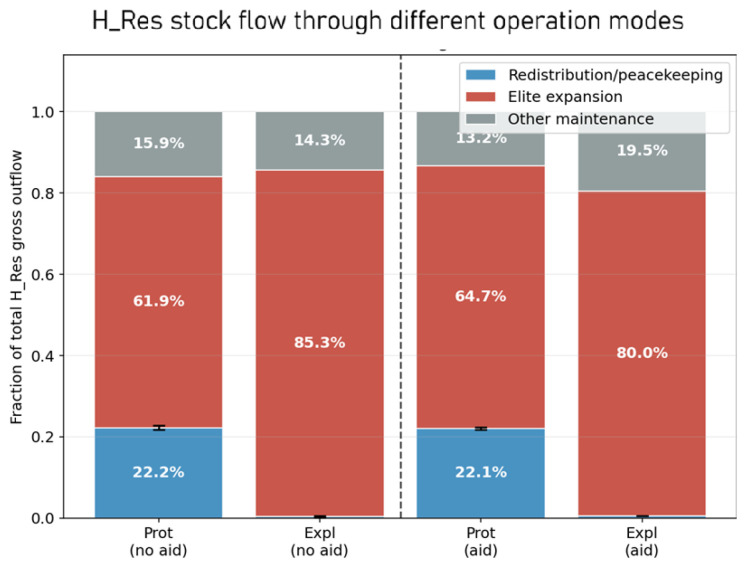
$H_{\mathrm{Res}}$ leverage analysis (N=10 seeds each). Stacked bars showing the fraction of total gross $H_{\mathrm{Res}}$ outflow ($r_{11}\times 1+r_{15}\times 1+r_{16}\times 1+r_{17}\times 1+r_{18}\times 2$) allocated to each destination category across all four scenarios. Error bars show ±1 SD on the redistribution segment (the key community-benefit fraction). Under protection, 22.6±0.6% of outflow reaches the community via redistribution and protective militarization; under exploitation, this fraction is 0.4±0.2%.

We depict in Figure 3 how $H_{\mathrm{Res}}$ flows through the different modes for the different strategies. Note that this is not an analysis of how the variables change over time, but of how they are used to sustain the dynamical process: For every unit of $H_{\mathrm{Res}}$ leaving the pool, the fraction that ever reaches the community is 22% but effectively 0% under exploitation—no matter how much aid is provided. In both regimes elite expansion absorbs the rest (∼63% in protection, nearly all in exploitation), but the decisive difference is the $r_{18}$ multiplier. Under protection it runs at 5 times larger, carving out the redistribution share. Under exploitation it is absent or negligible. From this analysis we argue that we can provide information to answer **Q3**: The $r_{18}$ multiplier is a fundamental parameter to create or destroy community’s share.

### 5.4. Projecting Modes of Operation over Different Stocks and Timescales

Figure 3 quantifies the stock $H_{\mathrm{Res}}$ allocation difference between the two strategies, but not how homogeneous that difference is, or at what temporal resolution the two strategies are distinguishable from process data alone. Answering this requires tracking mode activity as a function of both time and window size, which is the objective of **Q4**.

The analysis in previous section was performed for a single variable $H_{\mathrm{Res}}$ in an averaged fashion. The idea of monitoring flows and stocks can be generalized by monitoring across *process modes*—recurring coalitions of reactions that co-activate in characteristic proportions and collectively drive species concentrations in a qualitatively distinct direction. This idea of measuring dynamical activity is inspired in reaction network analysis in biochemistry for detecting stationary regimes [63,64]. We formalize here a process mode as a weighted process vector $d_{m}\in R^{22}$, whose non-zero entries identify the participating reactions and their relative intensities (Table 6). For illustrative purposes, we introduce five modes that span the principal dynamical tendencies of the system: community self-maintenance, conflict amplification, and two competing chiefdom strategies called protection and exploitation.

While the first two modes are internal community dynamics, the protection and exploitation modes close a community feedback loop supported by Chiefdom: In the protection mode, chiefdom extraction ($r_{12}$) produces $H_{\mathrm{Res}}$, redistribution ($r_{18}$) converts it back to $C_{\mathrm{Res}}$, which sustains surplus production ($r_{2}$) and displaced-person reintegration ($r_{5}$), which in turn sustains the collective *C* that feeds back into $r_{12}$. The chiefdom exploitation mode instead includes $r_{13}$ (direct community-resilience extraction: $H+C_{\mathrm{Res}}\to H_{\mathrm{Res}}$) and excludes $r_{18}$.

Note that a Mode of Operation represents a process vector that can be a quantitative realization of one or more feedback loops; it measures how many times each reaction fires, whereas a feedback loop is a qualitative causal circuit. Since modes of operation can operate at different timescales, let $n_{t}\in R^{22}$ be the reaction-firing count vector at step *t*. The causal cumulative process vector over a window of *w* steps is

(7) $$v\left(t,\;w\right)\;=\;\sum_{s\;=\;t-w+1}^{t}n_{s},$$

and the time-localised projection onto mode *m* is

(8) $$p_{m}\left(t,\;w\right)\;=\;{\hat{d}}_{m}\;\cdot \;v\left(t,w\right),$$

bounded in [0,||v||]. If we additionally divide such measure by ||v|| we obtain the *cosine similarity*, which measures the directional alignment between the mode and the process in a [0,1] normalized scale. Looking at only cosine would tell you the mode is dominant but not whether the system is actually doing much. The window size *w* controls which dynamical layer is visible: at small *w*, the projection captures individual reaction bursts and step-level stochastic fluctuations; at large *w*, the cumulation suppresses high-frequency noise and reveals the slow structural attractor the system is tracking.

**Figure 4:**
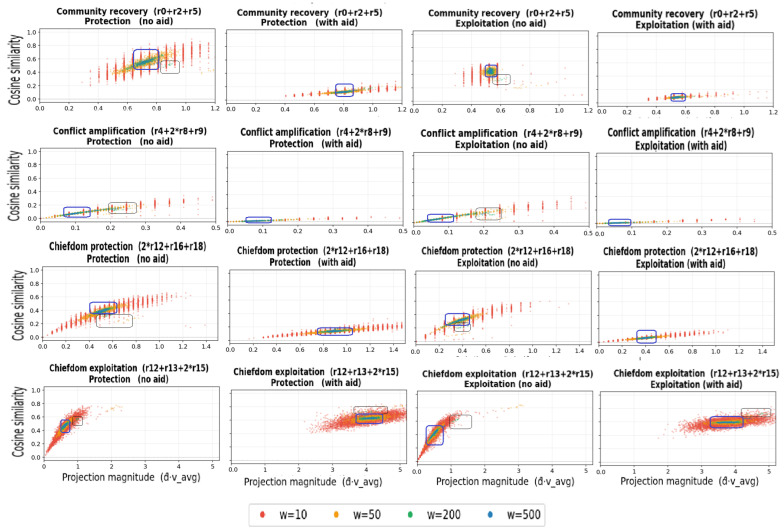
Mode-projection scatter for the five canonical modes without foreign aid ($F_{H}=0$). Each point represents one non-overlapping block of *w* steps; horizontal axis: projection magnitude ${\hat{d}}_{m}\;\cdot \;\bar{v}$; vertical axis: cosine similarity between the aggregate process vector and the mode direction. Color identifies window size (red w=10, orange w=50, green w=200, blue w=500). Under protection (**left**), activity concentrates in modes 1 and 3; under exploitation (**right**), in modes 2 and 4—consistent with the leverage outcomes shown in Figure 3.

Figure 4 shows the projection modes for the two strategies in both with and without aid cases. Each panel plots the projection magnitude ${\hat{d}}_{m}\;\cdot \;\bar{v}$ against the cosine similarity of the aggregate process vector with mode *m*, for non-overlapping blocks of width *w*. It reveals four distinct signatures across modes and scenarios that enable analyzing **Q3** from a flow-centered perspective: Community recovery, conflict amplification, and Chiefdom protection modes align between 2 and 5 times stronger with the process in the no-aid scenarios with respect to the aid scenario. The opposite occurs for the Chiefdom exploitation mode. Moreover, the chiefdom exploitation mode shows the largest absolute projections of all strategies; critically, the with-aid exploitation cloud extends visibly further right than the no-aid case, providing scatter-level evidence that aid amplifies the expansion cycle rather than redirecting it. Across all panels, cosine similarity transitions from diffuse at short windows (w=10) to highly concentrated at long windows (w=200,500), confirming that a modal signature is latent at fast timescales: We mark such clusters with an indigo box. At w=200 we observe a peculiar effect: a much smaller cluster typically having a larger projection magnitude than the major cluster. We mark such secondary clusters with a thin black box. This secondary cluster represents a different modal dynamic that we believe might trigger under certain conditions that could be related to stochastic increasings in grievances.

Therefore, by monitoring the conflict with an integration horizon with w=10,50, we observe both strategies as broadly equivalent. The distinction between a redistributive and an extractive chiefdom is a slow structural property–which motivates the multi-window classification below.

### 5.5. Sign-Based Mode Classification per Timescale

An alternative reading of the time-aggregated mode projections enables classifying the cumulative process by the sign of $S\;v_{w}\left(t\right)$ for species groups considered markers of specific phenomena within the dynamics. This enables considering **Q4** from a stock-centered point of view.

**Definition 1** (Process-mode classification)**.** *For a species group G and window w, let $\Delta x_{G}=\left(S\;v_{w}\left(t\right)\right){|}_{G}$. The window is classified* overproduction *if all entries of $\Delta x_{G}$ are strictly positive;* problem *if all are strictly negative;* challenge *if mixed signs;* steady state *if all are near zero.*

**Figure 5:**
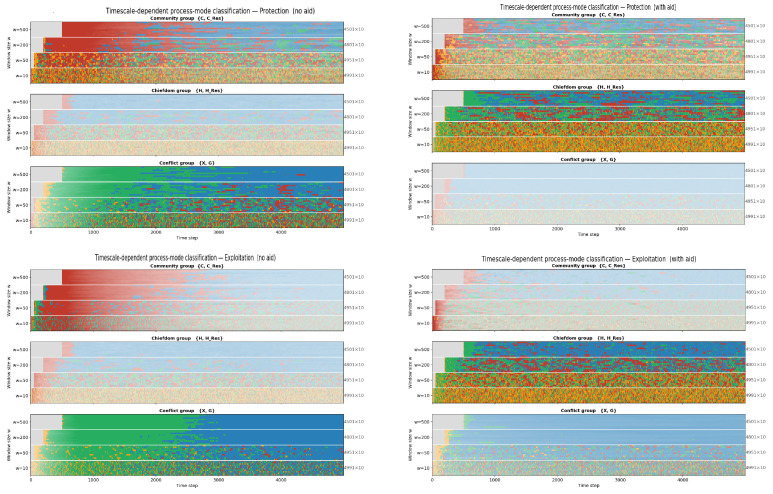
Timescale rasters for the four scenarios. **Top** row: protection without (**left**) and with (**right**) aid. **Bottom** row: exploitation without (**left**) and with (**right**) aid. Sub-panels classify community $\left\{C,C_{\mathrm{Res}}\right\}$, chiefdom $\left\{H,H_{\mathrm{Res}}\right\}$, and conflict {X,G} across w∈{10,50,200,500}. Blue: steady state. Green: overproduction. Yellow: challenge. Red: problem. Transparency indicates presence of the population stock associated with the group.

The classification is parameter-free and aligns with substantive groups: community $\left\{C,C_{\mathrm{Res}}\right\}$, chiefdom $\left\{H,H_{\mathrm{Res}}\right\}$, conflict {X,G}. Plotted as a raster with *w* vertical and time horizontal, it turns the trajectory into a structural map (Figure 5). Note that transparency is calculated as the relative proportion of the population stock associated with the stocks group in the plot.

By looking at the transparency of the plots in the different cases we confirm the dominant population groups as described in Table 5. Interestingly, the plot shows that a short time scale stochastic fluctuations make very difficult to read what is happening. In all plots the stochastic runs with w=10,50 look mostly as noise for all groups. When we grow the window size exploitation without aid registers *problem* for the community group at larger windows during the expansion phase, while protection registers *steady state* at large *w* even when shorter windows still classify it as *challenge*. For reasons of space we will not elaborate further on the many conclusions that can be advanced from this analysis, but we want to remark that this analysis enables characterizing the timescale at which the integrative and extractive chiefdom dynamics can be properly differentiated. This is the formal counterpart of the adaptive peacebuilding prescription for multi-horizon monitoring [7,55,62].

## 6. Chemical Organization Theory and the Statistical Geometry of Regimes

### 6.1. Organizations, Lattices, and the Conflict Model

The previous dynamical and stoichiometric analyses establish what a conflict system does under specific parameterizations: which trajectories emerge, which modes dominate, and at what timescale strategies become distinguishable. A complementary and more fundamental question is what the system is structurally capable of, independent of any particular choice of rate constants. When a system is too complex to trace dynamics through parameters, Chemical Organization Theory (COT) [20,50] provides the language for this question.

Given a reaction network (M,R), a subset O⊆M is a *chemical organization* if it satisfies two structural conditions simultaneously:

1. *closure*—no reaction whose reactants are all in O produces a species outside O; and
2. *self-maintenance*—there exists a strictly positive flux vector v>0 restricted to the reactions enabled by O such that the net production of every species in O is non-negative: Sv≥0.

Organizations are thus the subsets of the network that can in principle sustain themselves indefinitely. We now introduce an extension of our previous Chiefdom-Community model that includes a state with institutional power on top of the other two. This model is empirically coherent with the Lake Chad Basin conflict that contains national states whose institutional apparatus operates alongside chiefdom formations [1,55].

### 6.2. The Full Network: Adding the State

We extend the network of Section 5 with four state species and one external aid species:

- *I*—institutionalized people (state personnel);
- $I_{\mathrm{Res}}$—state-controlled resources;
- $P_{I}$—state military or police force;
- *L*—legitimacy, rule of law, infrastructure (the resource the state form most distinctively produces);
- $F_{I}$—foreign aid channeled to the state (catalytic).

**Figure 6:**
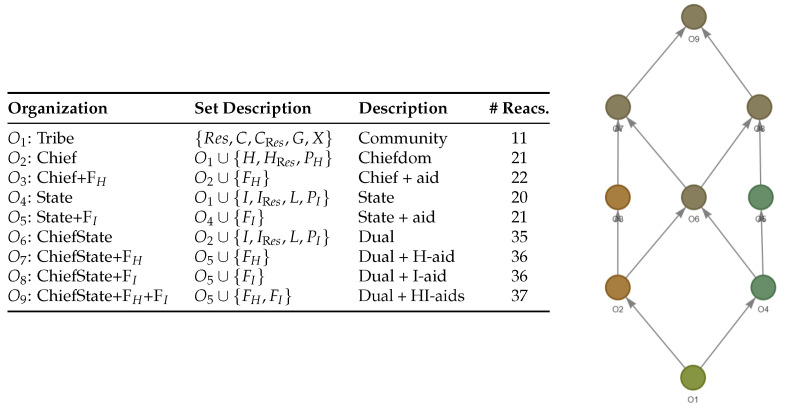
Hasse diagram of the 9 chemical organizations of the full conflict reaction network (14 species, 37 reactions). Arrows denote direct set inclusion the color represents the proportion of peace (green) vs. conflict (orange) species in the organization. Trivial organizations not containing population species or non-reactive species in the set are omitted.

The state subnetwork mirrors the chiefdom subnetwork in functional role but differs in mechanism: the state extracts via codified taxation requiring *L* as a catalyst, recruits institutional personnel from *C* and from the displaced *X* when *L* is sufficient, maintains a security force $P_{I}$ from $I_{\mathrm{Res}}$, and produces *L* from *I* and $I_{\mathrm{Res}}$. Legitimacy *L* decays autonomously and must be permanently reconstructed—the structural feature that distinguishes the state form from the chiefdom form. Table 7 summarizes the state and cross-regime reactions added on top of Table 4, and Figure 6 shows the hierarchy of organizations that is obtained for the extended reaction network.

Cross-regime reactions link the chiefdom and the state. Corruption ($r_{31}$) transfers $I_{\mathrm{Res}}$ to $H_{\mathrm{Res}}$; co-optation ($r_{32}$) runs in the opposite direction; mutual armed conflict ($r_{35}$) consumes $P_{H}$ and $P_{I}$ jointly; and the two foreign-aid catalysts $F_{H},F_{I}$ supply external inflows independently ($r_{21}$ and $r_{36}$).

### 6.3. Elementary Sustainable Modes of Operation

Organizations characterize the *potentially stable substructures* of a reaction network, but they say nothing about how the system *settles* into them or moves *between* them. To analyze these aspects we introduce the notion of **Elementary Sustainable Mode of Operation** (ESMO).

Note $O_{7}$ represents the organization where both Chiefdom and State co-exist without any external aid. Its stoichiometric matrix is $S\in R^{12\times 35}$. Admissible flux states are non-negative vectors $v\in R_{\geq 0}^{35}$, i.e., reactions can only fire forward. This defines the *positive orthant cone* $K=R_{\geq 0}^{35}$.

For a directed transition from an organizational regime *A* to a regime *B*, we define a *transition signature* $\sigma_{A\to B}\in {\left\{-1,\;0,\;+1\right\}}^{12}$ derived from the characteristic states of the two regimes. Let $x^{A}$ and $x^{B}$ be representative species-abundance vectors for regimes *A* and *B*, respectively. For each species *s*:

(9) σs=+1ifxsB−xsA>θ−1ifxsB−xsA<−θ+0otherwise,

where θ>0 is a neutrality threshold that filters species whose abundance difference is negligible relative to the regime contrast ($\theta =10^{-4}$ in our simulations). The signature enables encoding a *coverability constraint* in the sense of Petri nets [65]: species with $\sigma_{s}=+1$ must be produced in net excess (${\left[Sv\right]}_{s}>0$), and species with $\sigma_{s}=-1$ must be consumed in net excess (${\left[Sv\right]}_{s}<0$). The constrained cone for transition A→B is

(10) $$C_{A\to B}\;=\;\left\{v\in R_{\geq 0}^{35}\;|\;\sigma_{s}{\left[Sv\right]}_{s}>0\;\;\forall \;s:\;\sigma_{s}\neq 0\right\},$$

intersected with the simplex $1^{⊤}v=1$ to obtain a bounded feasible polytope $P_{A\to B}$. An ESMO is a **vertex** of $P_{A\to B}$: a minimal, non-decomposable reaction coalition that simultaneously satisfies all sign constraints. Vertices of a linear polytope are the elementary solutions from which all feasible flux states can be written as convex combinations; they are the analogous to elementary flux modes in metabolic stoichiometry [63], but instead of encoding steady states ESMO encodes transition directions.

### 6.4. Parameter-Free Regime Transition Probabilities Between Conflict and Peace States

The number of vertices $N\left(A\to B\right)=|\mathrm{vert}\left(P_{A\to B}\right)|$ measures the number of different ways in which the transition with signature $\sigma_{A\to B}$ can occur. Larger *N* means the transition is structurally richer, and $P_{A\to B}=\emptyset$ the transition is stoichiometrically infeasible regardless of kinetics [66]. In this way, ESMO distributions provide a principled basis for estimating transition probabilities among regimes without fitting kinetic parameters. Transitions only depend on knowing stocks, which is far easier to measure empirically (population counts, resource levels, institutional indicators) than knowing how reaction kinetics is parameterized.

In general, let $x_{1}^{A},\ldots ,x_{m}^{A}$ and $x_{1}^{B},\ldots ,x_{n}^{B}$ be distinct (linearly independent) instantiations of each regime, the pairwise ESMO computations produce a *distribution* of vertex counts over all m×n signature problems. We define the **structural feasibility probability**Pr(A→B) as follows:

(11) $$\mathrm{Pr}\left(A\to B\right)\;=\;\frac{\left|\{\left(i,j\right)\;:\;N\left(x_{i}^{A}\to x_{j}^{B}\right)>0\}\right|}{m\;\cdot \;n},$$

We now show an example comparing transitions between conflict (C) and peace (P) configurations for each organization. We define a library of m=n=5 representative states, so that different pairs (i,j) produce distinct LP sign patterns (signatures). We combined different values of high/low displaced and grievances, with variations on other stocks to illustrate different conflict and peace situations.

**Figure 7:**
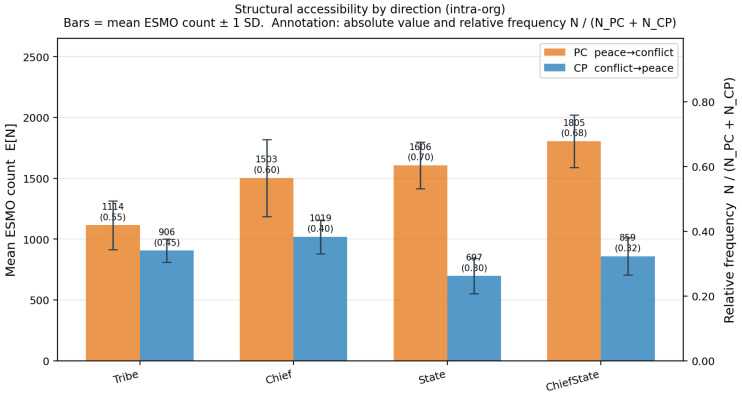
Mean ESMO count per direction and organization (bars ± 1 SD). Bar annotations give the absolute mean and the relative frequency $f=\bar{N}/\left({\bar{N}}_{\mathrm{PC}}+{\bar{N}}_{\mathrm{CP}}\right)$. The right axis shows the relative frequency scale. The systematic dominance of PC over CP, and its amplification with organizational complexity, is evident across all four regimes.

All m×n=25 PC pairs and all n×m=25 CP pairs are enumerated per organization. From the distribution of ESMO counts over all pairs, we report:

$${\bar{N}}_{\mathrm{PC}}\left(X\right)=\frac{1}{25}\sum_{i,j}N\left(x_{i}^{P}\to x_{j}^{C}\right);\;{\bar{N}}_{\mathrm{CP}}\left(X\right)=\frac{1}{25}\sum_{i,j}N\left(x_{i}^{C}\to x_{j}^{P}\right),$$

and the *relative frequency* of each direction:

(12) $${\mathrm{Pr}}_{\mathrm{PC}}\left(X\right)=\frac{{\bar{N}}_{\mathrm{PC}}}{{\bar{N}}_{\mathrm{PC}}+{\bar{N}}_{\mathrm{CP}}},\;{\mathrm{Pr}}_{\mathrm{CP}}\left(X\right)=\frac{{\bar{N}}_{\mathrm{CP}}}{{\bar{N}}_{\mathrm{PC}}+{\bar{N}}_{\mathrm{CP}}},$$

which can be interpreted as a probabilistic measure for each transition direction. Figure 7 reports the transition statistics for all four relevant organizations (we do not consider organizations with aid for simplicity). Interestingly, the number of conflict ESMOs grow monotonically with complexity, but that is not the case for recovery ESMOs. This is a first parameter-free topological result that explains conflict protractedness: more complex organizational forms have a progressively stronger structural bias toward transitioning from peace to conflict than in the reverse direction within the same organizational regime.

**Figure 8:**
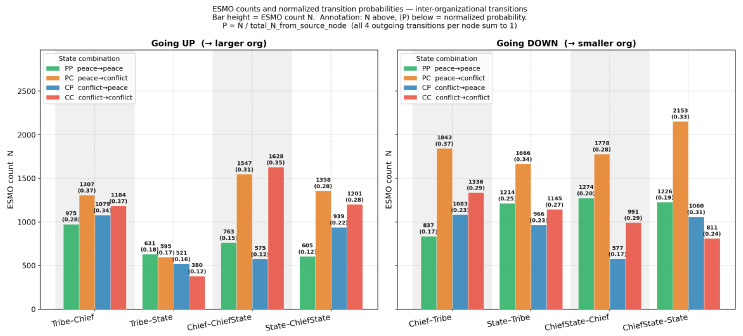
ESMO counts disaggregated by state-combination type (PP = peace→peace, PC = peace→conflict, CP = conflict→peace, CC = conflict→conflict) for each cover edge. **Left** panel: going up the lattice (→ larger organization); **Right** panel: going down. Bar height encodes the ESMO count; taller bars indicate greater structural richness.

We now extend our analysis by counting the number of ESMOs for every directed pair along the four cover edges of the sub-lattice $\left\{O_{1},O_{2},O_{4},O_{5}\right\}$, in both directions and across all four peace/conflict state combinations (PP, PC, CP, CC), yielding 32 structurally distinct LP problems in the 12-dimensional flux space of the $O_{5}$ sub-network (Chiefstate). Aggregated counts by transition type across all edges are summarised in Figure 8.

Our structural analysis reveals several interesting asymmetries both with respect to the transition direction and the conflict status of the endpoints. For example, Downward transitions (State → Tribe) are consistently flatter than upward (Tribe → State), except for the Chief-Chiefstate transition. For example, the two possible transitions from Peace to Peace and Peace to Conflict (green and yellow) for the upward Tribe-Chief transition (left plot) find relative frequencies 0.28 and 0.37, while the downward version find relative frequencies 0.17 and 0.37. By looking the other transitions we confirm the trend, but the Chief-Chiefstate transition is the exception. This exception is consequence of its significantly larger tendency to drive to conflict (yellow and red bars) in the upward direction with probabilties 0.31 and 0.35.

Transitions among organizations are strongly asymmetric: Under peace–peace (PP) the upward tribe-chiefdom is favored (0.28 vs. 0.17), while under peace–conflict (PC) the downward (chiefdom-tribe) are equal (0.37). Mixed (CP) and conflict–conflict (CC) cases are nearly symmetric as well. Following a similar analysis, we can conclude that the Tribe–Chief boundary is more bidirectionally permeable than the Tribe–State one, with directional advantage concentrated in peace–to–conflict transitions.

Concerning the kind of regime the transition is going through, we observe that organizational descent with conflict intensification is the richest. The global maximum (N=2153) is the peaceful ChiefState → State-conflict descent, followed by peaceful Chief → Tribal-conflict descent (N=1843). The global minimum (N=380) is the conflict-ridden Tribe → State-conflict ascent. State-building under conflict is the most structurally constrained scenario: assembling the institutional apparatus ($I,I_{\mathrm{Res}},L,P_{I}$) while simultaneously generating displaced (*X*) and grievances (*G*) poses incompatible stoichiometric requirements.

### 6.5. Structural Entropy and the Markovian Attractor Landscape

**Figure 9:**
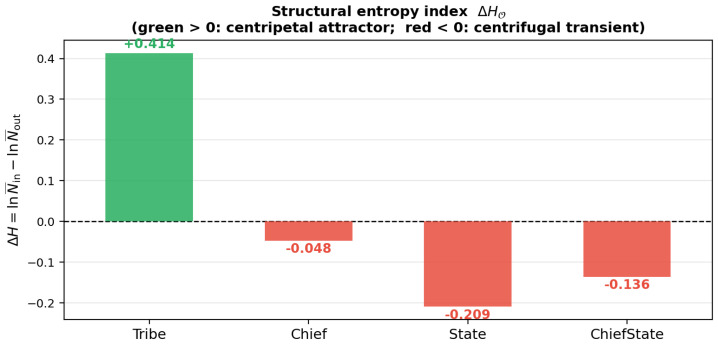
Structural entropy index $\Delta H_{O}=\ln{\bar{N}}_{\mathrm{in}}-\ln{\bar{N}}_{\mathrm{out}}$ for each regime. Green bar (ΔH>0): centripetal organizational attractor, with more incoming than outgoing structural pathways. Red bars (ΔH<0): centrifugal transients, with more outgoing than incoming pathways. Tribe is the sole centripetal regime; Chief, ChiefState, and State are all centrifugal under population-consistent normalization.

We define the structural entropy index $\Delta H_{O}=\log{\bar{N}}_{\mathrm{in}}\left(O\right)-\log{\bar{N}}_{\mathrm{out}}\left(O\right)$ to measure the extent at which an organization acts as a structural attractor (ΔH>0, centripetal) or a structural transient (ΔH<0, centrifugal), aggregating mean accessibility over all incoming and outgoing cover–edge transitions. Figure 9 shows the structural entropy of the organizations.

Computing $\Delta H_{O}$ from the 32 directed ESMO counts yields a clear hierarchy (Figure 9). A striking feature is that *all organizations except Tribe are centrifugal* (ΔH<0). Under population-consistent normalization, the organizational lattice is fundamentally asymmetric: only the minimal Tribe regime acts as a structural attractor, while Chief, ChiefState, and State all exhibit net structural outflow.

Tribe is the only centripetal organization (ΔH=+0.414). We link this to the difficulty of building institutional layers from a tribal start. Tribe is the unique structural attractor. Next, Chief is weakly centrifugal (ΔH=−0.048). It acts as a high-throughput conduit, equally connected upward to ChiefState and downward to Tribe, serving as an unstable transition hub rather than an attractor. Surprisingly, State is the most centrifugal organization (ΔH=−0.209), and ChiefState appears as centrifugal (ΔH=−0.136) but less than state.

**Figure 10:**
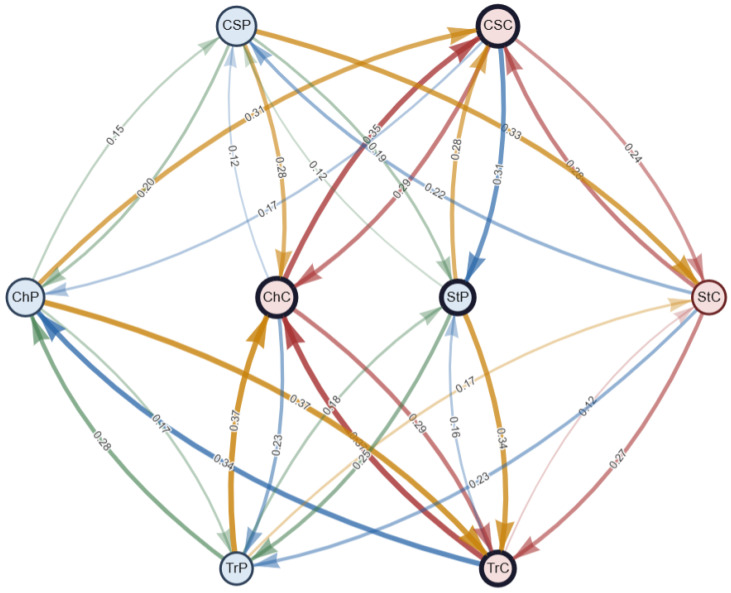
Structural Markov graph on the eight (org,state) nodes obtained by crossing the four organizational regimes (Tribe, Chief, State, ChiefState) with the two state types (Peace, Conflict).

By observing the Markov graph built from the probabilities in Figure 9 we can observe some important structural tendencies in this Markov graph that reveal documented dynamics in the Lake Chad Basin. Figure 10 shows such Markov Graph omitting the intra-organization transitions (self-transitions), which do not add information to the inter-organizational dynamics.

**(i) Peace and conflict are equally destabilizing at the tribal–chiefdom interface.**

From TrP the dominant transition is TrP→ChC (P=0.37), nearly identical to TrC→ChC (P=0.37). Tribal peace and tribal conflict are thus *equally likely* to generate chiefdom-level conflict. Conversely, ChP→TrC (P=0.37) is the dominant exit from chiefdom peace. Together, these probabilities describe a structural trap tribal level stabilises the chiefdom, and chiefdom peace tends to generate tribal conflict. In the Lake Chad Basin, this resonates with patterns in which inter-community ceasefires create resource-competition vacuums that chiefs cannot arbitrate, while chieftaincy disputes routinely re-ignite village-level violence.

**(ii) Conflict escalates upward and collapses back.**

The dominant 4-cycle TrC→ChC→CSC→StP→TrC traces a recurrent structural sequence: tribal conflict escalates to chiefdom conflict (P=0.37), which escalates further to the dual-hierarchy level (P=0.35), where a temporary state peace becomes accessible (P=0.31), but that peace structurally relapses into tribal conflict (P=0.34). This mirrors the documented cycle in the basin in which multinational-level interventions (MNJTF, Lake Chad Basin Commission) produce negotiated ceasefires that do not resolve community-level tensions, leaving displacement and armed-group reorganization at the tribal scale as the default outcome.

**(iii) Dual-hierarchy conflict is a self-reinforcing gateway.**

CSC exits most strongly to StP (P=0.31) but also back to ChC (P=0.29), creating an internal oscillation at the top of the governance hierarchy. State peace produced from dual-hierarchy conflict in turn flows back to CSC (P=0.28), suggesting that top-down peace settlements leave chiefdom–state tensions unresolved, perpetuating a high-level conflict loop even when tribal violence temporarily subsides.

## 7. Conclusions

We set out to explain why protracted conflicts persist for analogous reasons why living systems persist. Conflicts form a collective of stocks whose transformation structure enables their self-production. By introducing the reaction network formalism and its modeling of conflicts we provide a new way to answer questions about conflicts whose structure is too complex and require new approaches that link structure with dynamics (see Table 1). Reading Service’s typology as a vocabulary of coexisting regime forms, we showed that each named form—tribe, chiefdom, state—is a chemical organization in a single reaction network (Claim 1); that one organization admits several self-maintaining modes of operation, the redistributive and predatory chiefdoms being the clearest pair (Claim 2); and that the mixed chiefdom–state configuration empirically matching the Lake Chad Basin is itself an organization whose several operation modes jointly sustain the conflict (Claim 3). These are the formal counterpart of de Coning’s “self-sustaining path dependencies of violence” [7,21]: the path dependencies are organizations, and the modes that sustain them are computable. The decisive results; however, are structural and parameter-free. Counting the elementary sustainable modes of operation along the lattice’s cover edges reveals a systematic bias toward conflict: transitions into conflict regimes are consistently richer than transitions out (PC≫CP across all regimes), and the dual chiefdom–state form—a mere transient under peaceful conditions—becomes a conflict attractor, closing a structural conflict–peace loop in which peace appears only as a transient mediator of the reset from complex to simple conflict.

The methodological payoff is not only parameter-free but also compositional: each diagnosis names a specific reaction pathway whose activity is responsible for the observed consequence—precisely the form of result that policy reasoning needs when a system has too many parameters to search and too many trajectories to inspect [62]. This yields three concrete implications for complex conflict management. First, identify leverage points by tracking flows, not just volumes. Our stoichiometric flow-allocation analysis (Section 5.3) reveals which reactions act as structural bifurcations—where redirecting existing flows matters more than scaling them up. Practitioners can map their own intervention mechanisms onto reaction networks and compute where a small change in allocation rules (e.g., surplus redistribution vs. elite retention) produces qualitatively different regime outcomes, independent of knowing precise rates.

Second, identify scales for multi-horizon monitoring. The timescale projection method (Section 5.4 and Section 5.5) shows that the same process data can be classified as “challenge” at short windows and “steady state” at long windows—or vice versa, directly supporting adaptive peacebuilding’s call for monitoring across multiple temporal scales. Third, sequence interventions structurally. Chemical Organization Theory (Section 6) identifies which regime configurations are structural attractors (centripetal) and which are transients (centrifugal) based solely on the network’s transition geometry. Practitioners can thus prioritise rebuilding foundational organizations (e.g., community-level resilience) before attempting to install more complex institutional layers. These are not empirical claims about this particular simulation, but general properties of the reaction-network toolkit: it turns intervention design from a parameter-tuning problem into a structural diagnosis of where flows go, at what timescale, and in what sequence.

Similarly we identify some challenges that require future work. First, measuring abstract stocks (community resilience, elite surplus, legitimacy) requires proxy indicators; future work must develop and validate empirical measurement protocols for these species. Second, the reaction set is derived from Lake Chad; applying the framework to other protracted conflicts (Colombia, Yemen, Sahel) will require adapting the network to different political economies and historical trajectories. Third, stoichiometric feasibility (ESMOs) does not guarantee kinetic reachability; future research should bridge this gap by incorporating realistic rate constraints or strategic behavior into the diagnostics, and by treating interventions as explicit reactions that can be tested for their ability to shift the organization lattice toward the tribal attractor.

We hope this work advances towards the establishment of a structurally grounded, parameter-free toolkit for analyzing conflict persistence and intervention leverage points, and it provides the formal foundations for applying reaction-network methods to protracted conflict management.

## Figures and Tables

**Table 1:** Comparison of modeling traditions along key analytical dimensions. Our proposed novel framework in last row. • = strong, ∘ = partial, − = absent.

Approach	Structural Richness	Dynamical Precision	Process Ontology	Kinetic-Independent	Scales to Complexity
Differential equations	∘	•	−	−	−
Game theory	∘	∘	−	−	−
Agent-based models	•	•	−	−	∘
Network models	•	−	−	•	•
Dynamical Systems	∘	∘	−	∘	−
Stock-and-flow/CLD	∘	∘	∘	−	∘
**Reaction networks**	•	•	•	•	•

**Table 2:** The eleven reactions of the band–tribe. (·) denotes a catalytic species (present on both sides, net change zero).

ID	Reaction	Mechanism	Saturation
$r_{0}$	∅→Res	Environmental resource inflow	none
$r_{1}$	(C)+Res→(C)	Collective consumes resources	Res
$r_{2}$	$\left(C\right)+\mathrm{Res}+\left(C_{\mathrm{Res}}\right)\to \left(C\right)+2\;\left(C_{\mathrm{Res}}\right)$	Communal labor grows community resilience	Res
$r_{3}$	$C_{\mathrm{Res}}\to \mathrm{Res}$	Stored resilience released back to ambient stock	$C_{\mathrm{Res}}$
$r_{4}$	C→X	Collective members displace under stress	none
$r_{5}$	$X+C_{\mathrm{Res}}\to C$	Displaced reintegrated in community	none
$r_{6}$	2Res→∅	Excess of resources decay	none
$r_{7}$	$2C_{\mathrm{Res}}\to \emptyset$	Excess of resources decay	none
$r_{8}$	(X)+(C)+Res→(X)+(C)+G	Displaced scarcity produces grievances	Res
$r_{9}$	2G+C→X	Displacement by grievance accumulation.	none
$r_{10}$	G→∅	Grievance decay (forgetting)	none

**Table 3:** Stoichiometric matrix *S* of the band–tribe conflict model. Catalytic entries (species with zero net stoichiometric coefficient) are highlighted in blue. Reaction labels follow the mechanistic narrative: $r_{1}$–$r_{5}$ are subsistence/resilience/reintegration; $r_{6}$–$r_{7}$ are resource and resilience decay; $r_{8}$–$r_{9}$ are conflict/grievance dynamics; $r_{10}$ is grievance decay.

Species	$r_{0}$	$r_{1}$	$r_{2}$	$r_{3}$	$r_{4}$	$r_{5}$	$r_{6}$	$r_{7}$	$r_{8}$	$r_{9}$	$r_{10}$
*C*	0	0	0	0	−1	+1	0	0	0	0	0
*X*	0	0	0	0	+1	−1	0	0	0	1	0
Res	1	−1	1	−1	0	0	−2	0	−1	0	0
$C_{\mathrm{Res}}$	0	0	+1	−1	0	−1	0	−2	0	−1	0
*G*	0	0	0	0	0	0	0	0	+1	−2	−1

**Table 4:** Chiefdom and foreign-aid reactions with stochastic parameters. $\kappa_{j}$: base rate constant; $a_{j}^{\mathrm{prot}}$, $a_{j}^{\mathrm{expl}}$: strategy multipliers.

ID	Reaction	Mechanism	$\kappa_{j}$	$a_{j}^{\mathrm{prot}}$	$a_{j}^{\mathrm{expl}}$
$r_{11}$	$H+H_{\mathrm{Res}}\to H$	Chiefdom maintenance	0.020	1.0	1.0
$r_{12}$	$C+H+\mathrm{Res}\to C+H+H_{\mathrm{Res}}$	Extraction from shared pool	0.017	8.0	3.0
$r_{13}$	$H+C_{\mathrm{Res}}\to H+H_{\mathrm{Res}}$	Extraction from community	0.0125	1.0	10.0
$r_{14}$	H→C	Hierarchy decay	0.010	1.0	1.0
$r_{15}$	$C+H+H_{\mathrm{Res}}\to 2H$	Hierarchy expansion	0.030	5.0	8.0
$r_{16}$	$H+X+H_{\mathrm{Res}}\to H+P_{H}$	Militarization of displaced	0.001	8.0	0.5
$r_{17}$	$P_{H}+H_{\mathrm{Res}}\to P_{H}$	Police maintenance	0.020	1.0	1.0
$r_{18}$	$2H_{\mathrm{Res}}\to H_{\mathrm{Res}}+C_{\mathrm{Res}}$	Redistribution to community	0.010	5.0	0.05
$r_{19}$	$P_{H}+G\to P_{H}$	Grievance suppression	0.003	2.0	5.0
$r_{20}$	$P_{H}\to C$	Demobilization	0.002	1.0	0.1
$r_{21}$	$H+F_{H}\to H+F_{H}+H_{\mathrm{Res}}$	Foreign aid	0.050	1.0	1.0

**Table 5:** Simulation outcomes for different foreign aid and strategy scenarios.

Foreign Aid	Strategy	Population Result	Interpretation
Yes	Protection	H>C>X	Integration
Yes	Exploitation	H>X>C	Expulsion
No	Protection	H∼C>X	Adaptation
No	Exploitation	X>H>C	Collapse

**Table 6:** Representative process modes of the chiefdom model. Each mode is a weighted process vector $d_{m}\in R^{22}$. Key species effects are derived from the net stoichiometric direction $S\;d_{m}$; ↑/↓ denotes net production/consumption of the most affected species.

Mode	Process Vector	Key Species Effect	Meaning
Community recovery	$r_{0}+r_{2}+r_{5}$	↑C, ↓X,	Tribe integrates bands
Conflict amplification	$r_{4}+2r_{8}+r_{9}$	↑X, ↑G, ↓C	Community internal disputes
Chiefdom protection	$2r_{12}+r_{16}+r_{18}$	$↑C_{\mathrm{Res}}$, $↑P_{H}$, ↓Res	Protection strategy
Chiefdom exploitation	$r_{12}+r_{13}+2r_{15}$	↑H, ↓C, $↓C_{\mathrm{Res}}$	Exploitation strategy

**Table 7:** Reaction network for state–chiefdom dynamics.

ID	Reaction	Mechanism
$r_{22}$	$C+2\;\mathrm{Res}+L\;\to \;C+C_{\mathrm{Res}}+I_{\mathrm{Res}}+L$	State resource production from infrastructure
$r_{23}$	I+L+C→2I+L	State attracts collective people
$r_{24}$	$I+I_{\mathrm{Res}}\;\to \;I+L$	State builds infrastructure and legitimacy
$r_{25}$	$I+X+L\;\to \;I+P_{I}$	State military recruited from displaced
$r_{26}$	$P_{I}+I_{\mathrm{Res}}\;\to \;P_{I}$	State military consume state resources
$r_{27}$	$P_{I}\;\to \;C$	State military demobilizes
$r_{28}$	2L→⌀	Legitimacy requires permanent reconstruction
$r_{29}$	I→C	State people can revert to collective
$r_{30}$	$I+I_{\mathrm{Res}}+L+X\;\to \;I+C+L$	State brings displaced back to communities
$r_{31}$	$H+H_{\mathrm{Res}}+I+I_{\mathrm{Res}}\;\to \;2H+2H_{\mathrm{Res}}$	Corruption: Chiefdom co-opt state personnel
$r_{32}$	$I+I_{\mathrm{Res}}+H\;\to \;2I$	Co-optation: State absorb chiefdom leaders
$r_{33}$	$P_{H}+I+L\;\to \;P_{H}+X+G$	Chiefdom attacks state
$r_{34}$	$P_{I}+H\;\to \;P_{I}+C+G$	State attacks chiefdom
$r_{35}$	$P_{H}+P_{I}\;\to \;2X+G$	Mutual armed conflict
$r_{36}$	$I+L+F_{I}\;\to \;I+L+F_{I}+I_{\mathrm{Res}}$	Foreign aid to state

## Data Availability

All data can be accessed from https://github.com/tveloz/pyCOT/tree/master/projects/XCEPT_Article_1, accessed on 14 June 2026.

## Supplementary Materials

The following supporting information can be downloaded at: https://www.mdpi.com/article/10.3390/e28070754/s1.
