# Supplementary material for: Modeling and Simulating Complex Conflict Management Using Reaction Networks
Source: Entropy (Basel). 2026 Jul 1;28(7):754. doi: 10.3390/e28070754 (PMC13408802; doi:10.3390/e28070754)
Supplement: Supplementary file 1 [file entropy-28-00754-s001.zip › entropy-4149649-supplementary.pdf]

Modeling and Simulating Complex Conflict Management Using Reaction Networks

Tomas Veloz [1,*], Dirk Bruin [2] and Cedric De Coning [3]

Supplementary Material

This appendix documents all modelling choices, parameter values, and algorithmic decisions for the three analytical sections of the paper. The full source code is available in the accompanying repository; the three scripts `script_section3.py`, `script_section4.py`, and `script_section5.py` reproduce every figure directly.

S1. Section 3—Band-Tribe Conflict Dynamics

Reaction Network

The model consists of five species $\mathcal{S} = \{C, X, Res, C\_Res, G\}$ and eleven reactions r_0–r_{10}, loaded from `data/Basic_example.txt`. Table S1 lists each reaction, its biological interpretation, and its kinetic law.

Table S1. Band-tribe model reactions. MM = Michaelis-Menten saturation; MA = mass-action. α_S and α_R are scenario modifiers for solidarity and reactivity respectively.

ID	Stoichiometry	Interpretation	Kinetic law	Type
r_0	$\varnothing \to Res$	Constant resource inflow	κ_0	MA
r_1	$C + Res \to C$	Band consumption	$\kappa_1 \cdot C \cdot \frac{Res}{Res+K_{r1}}$	MM
r_2	$C + Res + C\_Res \to C + 2\,C\_Res$	Solidarity: surplus growth	$\alpha_S\,\kappa_2 \cdot C \cdot \frac{Res}{Res+K_{r2}} \cdot C\_Res$	MM
r_3	$C\_Res \to Res$	Resilience redistribution	$\kappa_3 \cdot \frac{C\_Res}{C\_Res+K_{r3}}$	MM
r_4	$C \to X$	Spontaneous displacement	$\kappa_4 \cdot C$	MA
r_5	$X + C\_Res \to C$	Solidarity: reintegration	$\alpha_S\,\kappa_5 \cdot X \cdot C\_Res$	MA
r_6	$2\,Res \to \varnothing$	Resource decay (second-order)	$\kappa_6 \cdot Res^2$	MA
r_7	$2\,C\_Res \to \varnothing$	Resilience decay (second-order)	$\kappa_7 \cdot C\_Res^2$	MA
r_8	$X + C + Res \to X + C + G$	Reactivity: grievance generation	$\alpha_R\,\kappa_8 \cdot X \cdot C \cdot \frac{Res}{Res+K_{r8}}$	MM
r_9	$2G + C \to X$	Reactivity: displacement by grievance	$\alpha_R\,\kappa_9 \cdot G^2 \cdot C$	MA
r_{10}	$2G \to \varnothing$	Grievance decay (second-order)	$\kappa_{10} \cdot G^2$	MA

ODE Kinetic Parameters

Table S2. Base rate constants and Michaelis-Menten saturation constants for the ODE.

Parameter	Value	Description
κ_0	0.200	Constant resource inflow
κ_1	0.020	Band consumption rate
κ_2	0.100	Surplus growth (scaled by α_S)
κ_3	0.010	Resilience redistribution
κ_4	0.010	Spontaneous displacement
κ_5	0.100	Reintegration (scaled by α_S)
κ_6	0.005	Res decay (second-order)
κ_7	0.010	*C_Res* decay (second-order)
κ_8	0.010	Grievance generation (scaled by α_R)
κ_9	0.050	Displacement by grievance (scaled by α_R)
κ_{10}	0.005	Grievance decay (second-order)
K_{r1}	3.0	MM saturation in r_1 (Res)
K_{r2}	3.0	MM saturation in r_2 (Res)
K_{r3}	2.0	MM saturation in r_3 (*C_Res*)
K_{r8}	3.0	MM saturation in r_8 (Res)

Two Scenarios

The solidarity modifier α_S scales reactions r_2 and r_5; the reactivity modifier α_R scales reactions r_8 and r_9. Two contrasting scenarios are compared:

- **Scenario A** (high solidarity, low reactivity): $\alpha_S = 2.0$, $\alpha_R = 0.5$.
- **Scenario B** (low solidarity, high reactivity): $\alpha_S = 0.3$, $\alpha_R = 3.0$.

Both scenarios share the initial condition $\mathbf{x}_0 = (C, X, Res, C\_Res, G) = (10, 3, 5, 2, 1)$.

ODE Integration

The ODE is integrated using `scipy.integrate.solve_ivp` with the LSODA method, tolerances `rtol` $= 10^{-8}$, `atol` $= 10^{-10}$, and evaluation at $N = 2000$ evenly spaced points on $[0, T_{\text{end}}]$ with $T_{\text{end}} = 400$. All denominators are protected by a floor of 10^{-9} to avoid division by zero.

Stochastic Simulation (Poisson Tau-Leaping)

The stochastic simulation uses Poisson tau-leaping with a fixed step size $\Delta t = 1.0$ for $N_{\text{steps}} = 300$ steps. The propensity of reaction j at state $\mathbf{x}$ is

$$\lambda_j = \kappa_j^{\text{stoch}} \cdot \pi_j(\mathbf{x}), \qquad \pi_j(\mathbf{x}) = \min_{s:\, S_{sj}^- > 0} \frac{x_s}{S_{sj}^-}, \tag{1}$$

where S_{sj}^- is the input stoichiometry (reactant coefficient) and π_j is the enabling degree (bottleneck reactant). At each step, firing counts are drawn as $n_j \sim \text{Poisson}(\lambda_j \Delta t)$, capped at $\lfloor \pi_j \rfloor$, and then rescaled by an iterative contention resolver (maximum 20 iterations) that prevents any species from going negative.

The stochastic rate constants κ^{stoch} differ from the ODE constants because the enabling-degree propensity underestimates multiplicative product kinetics. At initial conditions, the ratio of the ODE rate to the enabling degree can reach $6\times$ for three-reactant reactions such as r_2. Both parameter sets are independent calibrations of the same qualitative model.

Table S3. Stochastic rate constants κ^{stoch}. Modifier reactions carry the same α factors as the ODE.

Reaction	κ^{stoch}	Note
r_0	2.000	Source (no reactant; $\pi_j = 1$)
r_1	0.025	ODE/enabling ratio ≈ 1 at $\mathbf{x}_0$
r_2	0.500	Solidarity; $\pi = \min(C, Res, C\_Res) = 2$ at $\mathbf{x}_0$
r_3	0.020	
r_4	0.010	Matches ODE
r_5	0.100	Solidarity; $\pi = \min(X, C\_Res) = 2$ at $\mathbf{x}_0$
r_6	0.050	Second-order; $\pi = Res/2$
r_7	0.020	Second-order
r_8	0.050	Reactivity; $\pi = \min(X, C, Res) = 3$
r_9	0.100	Reactivity; $\pi = \min(G/2, C) = 0.5$
r_{10}	0.010	Grievance decay

S2. Section 4—Chiefdom Emergence and Strategy

Reaction Network

The chiefdom model extends the band-tribe network by adding a hierarchy species H, its resource pool H_Res, a police force P_H, and an external aid signal F_H (catalytic; never consumed). The full system has nine species

$$\mathcal{S} = \{C,\ X,\ H,\ Res,\ C\_Res,\ H\_Res,\ G,\ P_H,\ F_H\}$$

and twenty-two reactions r_0–r_{21} loaded from `data/Intrmediate_example.txt`. Reactions r_0–r_{10} are the band-tribe baseline (parameterised at Scenario A: $\alpha_S = 2$, $\alpha_R = 0.5$ baked into κ^{stoch}). Reactions r_{11}–r_{21} govern the chiefdom.

Initial Conditions

$$\mathbf{x}_0 = (C,\ X,\ H,\ Res,\ C\_Res,\ H\_Res,\ G,\ P_H,\ F_H) = (500,\ 50,\ 50,\ 10,\ 50,\ 100,\ 30,\ 10,\ 0).$$

The chiefdom starts as a small perturbation ($H = 50$, $H\_Res = 100$) on top of a harmonious band-tribe baseline.

Stochastic Simulation

Simulation uses Poisson tau-leaping with $\Delta t = 1.0$ and $N_{\text{steps}} = 5000$, with $N_{\text{seeds}} = 10$ independent replicates per scenario. Foreign aid F_H is injected adaptively: $F_H = 100$ whenever $x_X \geq 50$ or $x_G \geq 10$; otherwise $F_H = 0$ (aid decays immediately when conditions are not met).

Chiefdom Allocation Strategies

Eight chiefdom-controlled reactions are scaled by allocation multipliers $A \in \mathbb{R}^8_{>0}$ (index order: $r_{12}, r_{13}, r_{15}, r_{16}, r_{17}, r_{18}, r_{19}, r_{20}$):

Table S4. Chiefdom allocation multipliers for the two strategies. Bold entries indicate reactions that are central to the mode definition ($\times$ weight in mode vector shown in parentheses).

Reaction	Role	A_{protect}	A_{exploit}	Mode membership
r_{12}: $C + H + Res \to H\_Res$	Res extraction	**8.0**	3.0	Protection ($\times 2$); Exploitation ($\times 1$)
r_{13}: $H + C\_Res \to H\_Res$	*C_Res* extraction	1.0	**10.0**	Exploitation ($\times 1$)
r_{15}: $C + H + H\_Res \to 2H$	Hierarchy expansion	5.0	**8.0**	Exploitation ($\times 2$)
r_{16}: $H + X + H\_Res \to P_H$	Militarize displaced	**8.0**	0.5	Protection ($\times 1$)
r_{17}: $P_H + H\_Res \to P_H$	Police upkeep	1.0	1.0	(neutral)
r_{18}: $2H\_Res \to H\_Res + C\_Res$	Redistribution	**5.0**	0.05	Protection ($\times 1$)
r_{19}: $P_H + G \to P_H$	Grievance suppression	2.0	5.0	(background)
r_{20}: $P_H \to C$	Demobilize police	1.0	0.1	(background)

Mode Definitions

Five canonical process-vector modes are defined as directions in reaction-rate space. Each mode is a non-negative vector $\mathbf{d} \in \mathbb{R}^{22}_{\geq 0}$; entries are reaction weights:

Community recovery: $\mathbf{d}_{\text{comm}} = r_0 + r_2 + r_5$.
Self-reinforcing band-tribe loop: resource inflow $\rightarrow$ *C_Res* surplus $\rightarrow$ reintegration of displaced.

Conflict amplification: $\mathbf{d}_{\text{conf}} = r_4 + 2r_8 + r_9$.
Destabilising spiral: $C \rightarrow X$, grievance accumulation, and grievance-driven further displacement. r_8 is double-weighted because it drives both grievance accumulation and feeds r_9.

Chiefdom protection: $\mathbf{d}_{\text{prot}} = 2r_{12} + r_{16} + r_{18}$.
Fuel path ($2r_{12}$): double-weight Res extraction builds *H_Res*; r_{13} is *not* in this mode (no *C_Res* drain). Redistribution path (r_{18}, KEY): returns wealth to community. Protective-force path (r_{16}): absorbs displaced into police.

Chiefdom exploitation: $\mathbf{d}_{\text{expl}} = r_{12} + r_{13} + 2r_{15}$.
Dual extraction fuel ($r_{12} + r_{13}$) feeds hierarchy expansion cycle ($2r_{15}$): $C + H + H\_Res \rightarrow 2H$. r_{18} is suppressed; no wealth returns to the community.

Foreign aid: $\mathbf{d}_{\text{aid}} = r_{21}$.
Catalytic *H_Res* injection. Excluded from all projection computations so that the four main modes are comparable across aid and no-aid scenarios.

Mode Projection Analysis

For a non-overlapping window of w steps ending at step t, the per-step average process vector is

$$\bar{\mathbf{v}} = \frac{1}{w} \sum_{s=t-w+1}^{t} \mathbf{n}_s, \tag{2}$$

where $\mathbf{n}_s \in \mathbb{Z}^{22}_{\geq 0}$ is the reaction-firing count vector at step s. For mode $\mathbf{d}$ with unit vector $\hat{\mathbf{d}} = \mathbf{d}/\|\mathbf{d}\|$, two summary statistics are computed:

$$\text{proj} = \hat{\mathbf{d}} \cdot \bar{\mathbf{v}}, \qquad \text{cos-sim} = \frac{\hat{\mathbf{d}} \cdot \bar{\mathbf{v}}}{\|\bar{\mathbf{v}}\|}. \tag{3}$$

The projection magnitude measures absolute mode activity; the cosine similarity measures mode dominance relative to all other reactions firing simultaneously. The scatter plots in Section 4 show one point per non-overlapping block per seed for each window size $w \in \{10, 50, 200, 500\}$.

Timescale Mode Classification

Each species group is classified into one of four qualitative modes based on the net change of each species within the window:

- **problem** — all $\Delta x_s < 0$ (net decline);
- **overproduction** — all $\Delta x_s > 0$ (net growth);
- **challenge** — mixed (some species grow, some decline);
- **steady-state** — all $|\Delta x_s| < \tau_{\text{tol}}$ (near-zero change, $\tau_{\text{tol}} = 0.05$).

Three species groups are tracked: $M_P = \{C, C\_Res\}$ (community production), $M_H = \{H, H\_Res\}$ (chiefdom), $M_C = \{X, G\}$ (conflict indicators). Cell opacity in the raster figures encodes the primary species' share of the total human population $C + H + X$, with parameters $\alpha_{\min} = 0.25$, $\alpha_{\max} = 1.0$, and full opacity at a share ≥ 0.5.

H_{Res} Community-Benefit Routing

The routing fraction measures what fraction of total *H_Res* outflow benefits the community:

$$\rho = \frac{n_{16} \cdot 1 + n_{18} \cdot 2}{n_{11} \cdot 1 + n_{15} \cdot 1 + n_{16} \cdot 1 + n_{17} \cdot 1 + n_{18} \cdot 2}, \tag{4}$$

where n_j is the cumulative firing count of reaction j in the window and the integer weights are the *H_Res* input stoichiometry coefficients. $\rho = 1$ means all *H_Res* flows to redistribution and peacekeeping; $\rho = 0$ means all flows to elite maintenance or expansion. Summary statistics use $w = 200$ averaged over the last 50% of simulation steps.

S3. Section 5—Structural Markov Graph and ESMO Analysis

Overview

Section 5 analyses transitions between organisational states using Extreme Stochastic Mode Objects (ESMOs). An ESMO is a vertex of the polytope defined by all non-negative flow vectors $\mathbf{v} \geq \mathbf{0}$ that are consistent with the stoichiometry of a given transition signature. The number of ESMOs for a source–target state pair is used as a proxy for the structural accessibility of that transition.

Organisational Lattice

Four organisational types form a cover lattice under the inclusion relation:

$$\text{Tribe} \subset \text{Chief}, \quad \text{Tribe} \subset \text{State}, \quad \text{Chief} \subset \text{ChiefState}, \quad \text{State} \subset \text{ChiefState}.$$

Each organisation can be in peace (P) or conflict (C) state, yielding eight nodes: TrP, TrC, ChP, ChC, StP, StC, CSP, CSC. Inter-organisational transitions follow the four cover edges (*up* = smaller $\rightarrow$ larger org; *down* = larger $\rightarrow$ smaller), and each directed cover edge carries two *combo* types (PP/PC for peace sources; CP/CC for conflict sources).

ESMO Computation

For each (edge, direction, combo) triple, the LP solver enumerates all vertices of the transition-feasibility cone. Vertex enumeration is performed using pyCOT's `Persistent_Modules_Generator`, which builds the reaction network from the transition signature and calls an external vertex-enumeration routine. The output is the number of vertices N_{ESMO} — a count of structurally distinct pathways through which the transition can be realised.

Results are cached in `outputs/complex_COT_ESMO/transition_summary.csv` and `outputs/intra_org_esmo/esmo_cache_intra.csv` to avoid recomputation (which can take several hours). The cache files are read directly by `script_section5.py` to regenerate all figures.

Conditional Relative Frequency (f-value)

For a directed cover edge and a given source-state type, the conditional relative frequency is:

$$f(\text{combo}) = \frac{N_{\text{ESMO}}(\text{combo})}{N_{\text{ESMO}}(\text{combo}) + N_{\text{ESMO}}(\text{complement})}, \tag{5}$$

where the complement swaps only the target-state component (PP$\leftrightarrow$PC; CP$\leftrightarrow$CC). Thus $f(\text{PP}) + f(\text{PC}) = 1$ for peace sources and $f(\text{CP}) + f(\text{CC}) = 1$ for conflict sources on the same directed edge. Each node has two outgoing cover edges; summing f-values across both edges gives 2, not 1.

Normalised Transition Probability (P-value)

To obtain probabilities that sum to 1 per source node across all four outgoing transitions, we compute:

$$P(u \to v) = \frac{N_{\text{ESMO}}(u \to v)}{\sum\limits_{v' : (u,v') \in E} N_{\text{ESMO}}(u \to v')}, \tag{6}$$

where the denominator sums over all four outgoing directed transitions from node u. The P-values reported in the Markov graph and in the inter-organisational bar charts (Section 5, Figures 5–7) are all computed with this normalisation.

Markov Graph Visualisation

The Markov graph is rendered in two formats by `plot_markov_hierarchy.py`:

- An interactive HTML file (`markov_hierarchy.html`) using the PyVis library.
- A static PNG (`fig_markov_loops.png`) using Matplotlib.

Arrow properties are driven by the P-value via a single unified formula:

$$\text{line width} = 12\,P, \qquad \text{opacity} = 2.2\,P. \tag{7}$$

With $P \in [0.10, 0.40]$, this maps to line widths $[1.2, 4.8]$ and opacities $[0.22, 0.88]$. All 32 arrows use the same formula with curvature rad $= 0.15$; bidirectional pairs curve to opposite sides automatically because the arc direction is relative to the travel direction.

Arrow colours encode the combo type:

Combo	Direction	Colour
PP	peace → peace	forest green `#3D7A4A`
PC	peace → conflict	amber `#C8810A`
CP	conflict → peace	steel blue `#2563A6`
CC	conflict → conflict	brick red `#A83232`

The configuration variable `SHOW_VALUE = 'P'` controls which value appears as edge labels (options: `'P'`, `'f'`, or `'both'`). The variable `WEIGHT_COL = 'P'` independently controls which value drives arrow thickness and opacity.

Dominant Structural Cycle

Greedy path-tracing from TrC (the most conflict-prone tribal node) following the maximum-P outgoing transition at each step reveals the dominant 4-cycle:

$$\text{TrC} \xrightarrow{P=0.344,\ \text{PC}} \text{ChC} \xrightarrow{P=0.374,\ \text{CC}} \text{CSC} \xrightarrow{P=0.352,\ \text{CC}} \text{StP} \xrightarrow{P=0.308,\ \text{CP}} \text{TrC}. \tag{8}$$

This cycle captures a structural attractor in which tribal conflict escalates through hierarchical layers before a fragile state-level peace formation collapses back to tribal conflict.
